# Comparison of Demographic and Clinical Characteristics of Taiwan Biobank Participants With Nonparticipants

**DOI:** 10.2188/jea.JE20240297

**Published:** 2025-04-05

**Authors:** Chi-Shin Wu, Le-Yin Hsu, Chen-Yang Shen, Wei J. Chen, Mei-Chen Lin, Chun Chieh Fan, Shi-Heng Wang

**Affiliations:** 1 National Center for Geriatrics and Welfare Research, National Health Research Institutes, Zhunan, Taiwan; 2 Department of Psychiatry, National Taiwan University Hospital, Yunlin Branch, Douliu, Taiwan; 3 Institute of Epidemiology and Preventive Medicine, College of Public Health, National Taiwan University, Taipei, Taiwan; 4 Graduate Program of Data Science, National Taiwan University and Academia Sinica, Taipei, Taiwan; 5 Institute of Biomedical Sciences, Academia Sinica, Taipei, Taiwan; 6 College of Public Health, China Medical University, Taichung, Taiwan; 7 Center for Neuropsychiatric Research, National Health Research Institutes, Miaoli, Taiwan; 8 Center for Population Neuroscience and Genetics, Laureate Institute for Brain Research, Tulsa, Oklahoma, USA; 9 Department of Radiology, University of California, La Jolla, California, USA; 10 Department of Medical Research, China Medical University Hospital, China Medical University, Taichung, Taiwan

**Keywords:** healthy volunteer effect, biobank, Taiwan biobank, representativeness, sociodemographic characteristics, clnical characteristics

## Abstract

**Background:**

This study investigated fundamental demographic variables within the Taiwan Biobank (TWBB) and compared them with national demographic statistics. Additionally, a matched cohort analysis compared TWBB participants with nonparticipants to uncover disparities in sociodemographic and clinical characteristics.

**Methods:**

A total of 128,663 individuals aged 30 to 70 years without cancer were recruited within the TWBB, and 514,652 nonparticipants matched by age and sex were randomly selected from the National Health Insurance claims database. Sociodemographic variables, healthcare utilization metrics, underlying medical conditions, and subsequent mortality and cancer risk were analyzed.

**Results:**

TWBB participants were more likely to be female, older, married, higher educated, with higher incomes, and urban residency. Healthcare utilization metrics showed minimal differences. Pre-cohort entry, TWBB participants had a higher prevalence of certain medical conditions, such as peptic ulcer disease, osteoarthritis, osteoporosis, and uterine leiomyoma in females. During follow-up periods, elevated mortality rates were observed among TWBB participants but decreased cancer risk.

**Conclusion:**

The TWBB cohort exhibits disparities in sociodemographic and health-related attributes compared to the general population, comprising participants who were older, female, married, higher educated, higher income, and predominantly resided in urban areas. While mortality rates are slightly elevated within the TWBB cohort, cancer incidence rates are lower. Despite limitations in representativeness, the TWBB’s size and exposure measures offer valuable insights into associations between exposures and health conditions.

## INTRODUCTION

The comprehensive collection of health-related information holds significant importance for advancing biomedical research and the evolution of precision medicine. The Taiwan Biobank (TWBB), launched in 2012, stands as a pivotal initiative in this domain, with its primary objective being the improvement of health outcomes and the construction of a population-specific reference of whole-genome genotyping of the Taiwanese populace while fostering advancements in biomedical research.^1^ Alongside genetic information, the TWBB meticulously compiles self-reported medical conditions, lifestyle patterns, and environmental risk factors, contributing extensively to the wealth of available data for scientific inquiry.^2^ Numerous significant studies have been conducted using the Taiwan Biobank.^3^^–^^7^

While the TWBB represents a sizable cohort, concerns persist regarding its generalizability. Previous research has pinpointed several determinants influencing individuals’ willingness to engage in biobanking initiatives. These factors encompass higher education levels^8^^,^^9^ and marital status.^10^ Nevertheless, findings regarding age and gender exhibit inconsistent associations with participation.^8^^,^^11^^,^^12^ Analyzing those who have participated in the United Kingdom Biobank, a “healthy volunteer effect”,^13^ indicating that participants tend to be healthier, was observed.^14^ The United Kingdom Biobank participants tend to skew towards older age, female gender, and residence in socioeconomically advantaged areas compared to nonparticipants. Moreover, they exhibit lower rates of obesity, smoking, and daily alcohol consumption, along with fewer self-reported health issues when juxtaposed with the general populace. Additionally, participants’ all-cause mortality and total cancer incidence are notably lower than those observed in the broader population.^14^

Disparities exist in the enrollment methodologies between United Kingdom and Taiwan Biobank. In the United Kingdom Biobank, participants are chosen and enrolled from the general population aged between 40 and 69 years using postal invitations and advertisements. Nevertheless, the TWBB recruitment strategy incorporates a community-based approach, enrolling men and women aged 30–70 years with no prior cancer diagnosis from over 30 recruitment sites located in medical centers or regional hospitals across Taiwan, strategically distributed according to population density across different counties and cities. Given the differing methodologies employed in participant enrollment, our investigation seeks to determine the presence of a healthy volunteer effect within the TWBB.

This study aimed to understand the fundamental demographic variables within the TWBB and compare them with basic demographic statistics. Additionally, we undertake a matched cohort analysis comparing TWBB participants and nonparticipants by leveraging linkage to the National Health Insurance (NHI) program. Through this analysis, we seek to elucidate disparities in demographic and social characteristics, medical diseases before and after cohort entry, and the risk of cancer incidence and mortality between the two groups.

## METHODS

### Data source

The TWBB has been actively recruiting study participants and undertaking extensive data collection endeavors, encompassing genetic data and regular assessments of self-reported medical conditions. To qualify for participation, individuals must be between the ages of 30 and 70 years and have no history of cancer. Additionally, each participant must provide informed consent, explicitly consenting to data linkage with external databases, such as Taiwan’s NHI claims database.

Taiwan’s NHI program was established in 1997 as a comprehensive and compulsory program covering 99% of Taiwan’s population of 23.5 million. This NHI claims database, derived from the NHI program, provides detailed information on demographics, clinical diagnoses, medical procedures, and prescription records.^15^ The accuracy of clinical diagnoses within the NHI research database (NHIRD) has been validated for various conditions, including common ailments and severe diseases, such as epilepsy, ischemic stroke, hypertension, diabetes, hyperlipidemia, atrial fibrillation, all types of cancer, and major psychiatric disorders, showing favorable levels of sensitivity and positive predictive values.^15^^,^^16^

The recruitment and sample collection procedures have received approval from the Internal Review Board of the Taiwan Biobank. Moreover, this study has been approved by the Central Regional Research Ethics Committee of the China Medical University in Taichung, Taiwan (approval number: CRREC-108-30).

### Study sample

Our study specifically targeted participants within the TWBB cohort, initially encompassing 131,834 individuals. Despite the inclusion criteria specifying no history of cancer, some individuals were identified with cancer-related claims in the NHI database. Following excluding individuals with cancer-related claims in the NHI database before the cohort entry date, totaling 3,171 cases (2.4% of the original sample), the resulting sample comprised 128,663 cases.

We used data from the Registry for Beneficiaries, a subset of NHIRD, and randomly selected four comparison individuals from this registry for each TWBB participant. These comparisons were 1:4 matched based on sex and birth year at the cohort entry date; none had a previous cancer diagnosis. In total, we identified 514,652 such comparisons. The cohort entry date for the comparison individuals was aligned with that of their corresponding TWBB participants.

### Characteristics and health outcomes

Socioemographic variables encompassed sex, age, marital status, educational level, monthly income, urbanization of residency, and healthcare utilization metrics, such as the number of outpatient visits and hospitalizations.

We assessed comorbid medical conditions within 2 years before and after cohort entry, encompassing 48 diseases across all major organ systems. Each condition required at least two outpatient or one inpatient claim for inclusion. Detailed information on the International Classification of Diseases code is present in eTable 1. After cohort entry, we measured the risk of all-cause mortality using the death certification database and the incidence of overall and specific cancers using the registry for catastrophic illness patients.

### Statistical analysis

We first compared the distribution of sex, age, marriage status, and education attainment in the TWBB participants with the general population using 2012 Census data and 2022 Census data. Descriptive analyses for monthly income, residence urbanization, outpatient visits, and hospitalization were conducted for the baseline characteristics of TWBB participants and nonparticipant comparisons. The differences in health conditions prior to cohort entry and subsequent risk of health conditions between the two cohorts were compared. Even slight deviations can yield statistically significant *P*-values in large sample sizes, potentially lacking practical significance. We employed a threshold of a standardized mean difference greater than 0.1 to pinpoint clinically meaningful differences.

Cox regression models were conducted to assess the hazard ratios (HRs) of TWBB participants regarding cancer incidence and mortality compared to nonparticipants. This analysis included both overall cancer risk and the risk for common types of cancer. The study followed up all participants from their cohort entry date until the occurrence of the outcome event or the end of 2020, whichever came first. All statistical analyses were performed using the SAS statistical package (version 9.4, SAS Institute Inc., Cary, NC, USA).

## RESULTS

We observed that individuals in the TWBB cohort were more likely to be female, older, married, and highly educated compared to the general population (eTable 2). When matched for age and sex, TWBB participants exhibited higher monthly incomes and were more likely to reside in urban areas. Regarding healthcare utilization, there were generally no discernible differences in the number of outpatient visits or the percentage of hospitalizations between TWBB participants and the comparison group (Table 1).

**Table 1.** Baseline characteristics of Taiwan Biobank participants and age- and sex-matched comparisons

	TWBB participants (*n* = 128,663)	Comparisons (*n* = 514,652)	SMD
Sex male, *n* (%)	46,102 (35.83)	184,408 (35.83)	0.000
Age, years, mean (SD)	50.20 (10.97)	50.20 (10.97)	0.000
Age groups, years			
30–39	29,644 (23.04)	118,576 (23.04)	0.000
40–49	31,189 (24.24)	124,576 (24.24)	0.000
50–59	38,019 (29.55)	152,076 (29.55)	0.000
60–70	29,811 (23.17)	119,244 (23.17)	0.000
Year at recruitment^a^			
≤2013	13,284 (10.32)	53,136 (10.32)	0.000
2014	15,972 (12.41)	63,888 (12.41)	0.000
2015	23,228 (18.05)	92,912 (18.05)	0.000
2016	19,569 (15.21)	78,276 (15.21)	0.000
2017	12,346 (9.60)	49,384 (9.60)	0.000
2018	17,730 (13.78)	70,920 (13.78)	0.000
2019	15,374 (11.95)	61,496 (11.95)	0.000
2020	11,160 (8.67)	44,640 (8.67)	0.000
Monthly income			
0 or missing	2,587 (2.01)	17,412 (3.38)	−0.085
<30,000	22,134 (17.20)	140,781 (27.35)	−0.246
30,000–44,999	39,256 (30.51)	160,413 (31.17)	−0.014
45,000–59,999	26,016 (20.22)	86,423 (16.79)	0.088
≥60,000	38,670 (30.06)	109,623 (21.30)	0.202
Urbanization			
Urban	73,972 (57.49)	276,969 (53.82)	0.074
Suburban	44,829 (34.84)	183,494 (35.65)	−0.017
Rural	9,862 (7.66)	54,189 (10.53)	−0.100
Number of outpatient visits per year before the cohort entry date			
<10	68,481 (53.23)	282,912 (54.97)	−0.035
10–19	39,261 (30.51)	142,124 (27.62)	0.064
≥20	20,921 (16.26)	89,616 (17.41)	−0.031
Hospitalization before the cohort entry date			
No	112,793 (87.67)	448,050 (87.06)	0.018
Yes	15,870 (12.33)	66,602 (12.94)	−0.018

SMD, standardized mean difference; TWBB, Taiwan Biobank.

^a^Year at recruitment for the comparison group indicates the year they were included in our analysis.

The prevalence of health conditions prior to cohort entry and subsequent risk of health conditions is present in Table 2. Before cohort entry, there were no clinically meaningful differences in health conditions, except for a higher prevalence of peptic ulcer disease, osteoarthritis, and osteoporosis among TWBB participants. Additionally, among female participants, uterine leiomyoma was more common in the TWBB group. There were no overt differences regarding subsequent risks of medical conditions, with SMDs also below 0.1 for all health conditions analyzed.

**Table 2.** Prevalence (%) of health conditions in the two years before cohort entry and the subsequent risk of health conditions among Taiwan Biobank participants, compared to age- and sex-matched controls

	Health conditions in the two years before cohort entry			Health conditions in the two years after cohort entry		
	TWBB participants (*n* = 128,663)	Comparisons (*n* = 514,652)	SMD	TWBB participants (*n* = 128,663)	Comparisons (*n* = 514,652)	SMD
Type I diabetes mellitus	0.36	0.49	−0.020	0.05	0.10	−0.018
Type II diabetes mellitus	13.54	14.66	−0.032	4.16	4.92	−0.037
Hyperlipidemia	30.43	27.32	0.069	9.42	9.29	0.004
Dementia	0.4	0.39	0.002	0.49	0.55	−0.008
Substance use disorders	2.95	3.78	−0.046	0.91	1.48	−0.052
Schizophrenia	0.31	0.64	−0.048	0.08	0.14	−0.018
Bipolar disorder	1.38	1.31	0.006	0.27	0.28	−0.002
Depressive disorder	11.1	9.13	0.065	2.18	2.01	0.012
Obsessive-compulsive disorder	0.42	0.37	0.008	0.07	0.05	0.008
Anxiety disorder	23.05	20.09	0.072	4.78	4.66	0.006
Attention deficit-hyperactivity disorder	0.17	0.11	0.016	0.03	0.01	0.014
Eating disorder	0.17	0.14	0.008	0.03	0.02	0.006
Somatoform	1.31	1.17	0.013	0.25	0.32	−0.013
Adjustment reaction	3.1	2.09	0.064	0.99	0.84	0.016
Parkinson’s disease	0.23	0.28	−0.010	0.22	0.26	−0.008
Multiple Sclerosis	0.02	0.01	0.008	0.02	0.01	0.008
Epilepsy	0.89	1.05	−0.016	0.20	0.27	−0.014
Migraine	5.86	5.12	0.032	0.72	0.79	−0.008
Extrapyramidal and movement disorders	2.06	1.92	0.010	0.69	0.70	−0.001
Sleep apnea	0.83	0.42	0.052	0.72	0.44	0.037
Sleep disturbance	27.07	24.85	0.051	6.34	6.28	0.002
Valvular heart disease	7.18	5.7	0.060	1.33	1.26	0.006
Hypertension	22.09	25.6	−0.082	6.43	7.59	−0.045
Coronary artery disease	12.62	11.38	0.038	3.57	3.47	0.005
Cardiomyopathy	0.16	0.21	−0.012	0.08	0.11	−0.010
Arrhythmia	10.33	8.39	0.067	2.26	2.27	−0.001
Stroke	5.91	5.73	0.008	1.85	1.95	−0.007
Atrial fibrillation	0.6	0.63	−0.004	0.47	0.51	−0.006
Chronic obstructive pulmonary disease	15.52	12.22	0.096	1.87	2.05	−0.013
Asthma	11.32	10.68	0.020	2.06	2.28	−0.015
Gastroesophageal reflux disease	8.93	6.6	0.087	8.79	7.03	0.065
Peptic ulcer disease	34.57	28.83	0.124	8.10	7.28	0.031
Irritable bowel syndrome	1.83	1.44	0.031	1.87	1.38	0.039
Ulcerative colitis	0.35	0.28	0.012	0.05	0.05	0.000
Crohn’s disease	2.09	2.41	−0.022	0.04	0.07	−0.013
Rheumatoid arthritis	3.07	2.37	0.043	0.58	0.54	0.005
Gout	11.49	10.74	0.024	1.59	1.95	−0.027
Osteoarthritis	30.38	23.79	0.149	7.64	7.20	0.017
Osteoporosis	7.75	4.81	0.121	2.13	1.46	0.050
Chronic kidney disease	2.06	2.34	−0.019	1.60	1.98	−0.029
Renal stone	11.64	10.57	0.034	2.30	2.51	−0.014
Congenital heart disease	0.29	0.27	0.004	0.16	0.13	0.008
Self-harm behaviors	0.22	0.3	−0.016	0.04	0.06	−0.009

	Female TWBB participants^a^ (*n* = 82,561)	Female Comparisons (*n* = 330,244)	SMD	Female TWBB participants^a^ (*n* = 82,561)	Female Comparison (*n* = 33,0244)	SMD

Dysmenorrhea	8.91	7.69	0.044	0.55	0.65	−0.013
Uterine adenomyosis	8.08	6.35	0.067	1.19	1.17	0.002
Endometriosis external	5.67	4.56	0.050	0.63	0.62	0.001
Uterine leiomyoma	23.99	18.87	0.125	3.17	3.13	0.002
Polycystic ovary syndrome	2.92	2.27	0.041	0.21	0.21	0.000

SMD, standardized mean difference; TWBB, Taiwan Biobank.

^a^Since these are gynecological disorders, only female participants were assessed.

The results for comparing cancer and mortality risk among TWBB participants and matched samples are present in Table 3. The mortality rate was elevated among TWBB participants compared to the matched comparisons (HR 1.35; 95% confidence interval [CI], 1.26–1.45). However, TWBB participants exhibited a decreased risk of subsequent all cancers (HRs 0.87; 95% CI, 0.84–0.91). However, this finding varied for specific types of cancers. There were no significant differences in the risks for pancreatic cancer, lung cancer, kidney cancer, hematologic malignancies, breast cancer, or gynecological cancers between the two groups. Notably, the risk for prostate cancer was higher in the TWBB cohort compared to nonparticipants.

**Table 3.** Comparing cancer and mortality risk among Taiwan Biobank participants and age- and sex-matched comparisons

	TWBB participant *N* = 128,663			Comparison *N* = 514,652				
	Event number	PY	Incidence (per 10,000 PY)	Event number	PY	Incidence (per 10,000 PY)	HR (95% CI)	*P*-value
**Mortality**	985	553,906	17.78	3,208	2,217,401	14.47	1.35 (1.26–1.45)	<0.001
**Cancer**	2,874	546,945	52.55	13,309	2,187,463	60.84	0.87 (0.84–0.91)	<0.001
Oropharyngeal cancer	135	553,577	2.44	986	2,215,025	4.45	0.63 (0.53–0.75)	<0.001
Esophageal cancer	22	553,870	0.40	271	2,217,017	1.22	0.38 (0.25–0.59)	<0.001
Stomach cancer	79	553,726	1.43	409	2,216,576	1.85	0.76 (0.59–0.97)	0.03
Colorectal cancer	315	553,100	5.70	1,680	2,213,226	7.59	0.77 (0.68–0.87)	<0.001
Liver cancer	180	553,504	3.25	1,209	2,215,421	5.46	0.66 (0.56–0.77)	<0.001
Pancreatic cancer	70	553,850	1.26	314	2,217,113	1.42	0.89 (0.68–1.16)	0.37
Lung cancer	443	552,983	8.01	1,782	2,214,231	8.05	1.01 (0.91–1.12)	0.91
Breast cancer^a^	660	356,694	18.50	2,763	1,427,236	19.36	0.92 (0.85–1.01)	0.07
Gynecological cancer^a^	242	357,865	6.76	1,072	1,431,912	7.49	0.90 (0.78–1.03)	0.13
Prostate cancer^a^	194	194,974	9.95	521	781,559	6.67	1.38 (1.17–1.63)	<0.001
Bladder cancer	50	553,777	0.90	300	2,216,625	1.35	0.70 (0.52–0.95)	0.02
Kidney cancer	69	553,721	1.25	342	2,216,646	1.54	0.80 (0.62–1.04)	0.10
Hematologic malignancy	114	553,626	2.06	396	2,216,524	1.79	1.15 (0.93–1.42)	0.19

CI, confidence interval; HR, hazard ratio; PY, person-year; TWBB, Taiwan Biobank.

^a^Incidence and hazard ratios for breast and gynecological cancers were estimated for women, and for prostate cancer, they were estimated for men.

## DISCUSSION

In the TWBB cohort, females, older individuals, married individuals, those with higher education and incomes, and those with urban residency were predominant. Healthcare utilization between TWBB and comparison groups showed no significant differences in outpatient visits or hospitalizations. Pre-cohort entry, TWBB participants had similar health conditions except for elevated rates of peptic ulcer disease, osteoarthritis, osteoporosis, and uterine leiomyoma in females. Nevertheless, TWBB participants exhibited higher mortality rates yet lower cancer incidence when compared to matched populations.

The TWBB participants, in line with United Kingdom Biobank,^14^ tended to be older and predominantly female compared to the general population. Moreover, TWBB participants are more likely to have higher incomes and reside in urban areas. While this demographic composition may not perfectly reflect the general population, our analysis revealed that the prevalence of most diseases among TWBB participants was generally comparable to that of nonparticipants.

Our findings revealed a higher mortality rate among TWBB participants compared to the general population. This contrasts with the “health volunteer effect” seen in other studies, such as the United Kingdom Biobank, where all-cause mortality was reduced by 46.2% in men and 55.5% in women aged 70–74 years, and cancer incidence rates were 11.8% lower in men and 18.1% lower in women.^14^ Similarly, Finnish health surveys found that nonparticipants had mortality rates twice as high in men and 2.5 times higher in women compared to participants.^17^

The absence of the health volunteer effect in the Taiwan Biobank may be attributed to its enrollment strategies. With 30 recruitment centers in medical centers or regional hospitals, enrollment was more inclined toward individuals with preexisting health concerns. Notably, our analysis revealed that participants in the TWBB exhibited a higher prevalence of conditions such as peptic ulcer disease, osteoarthritis, osteoporosis, and uterine leiomyoma among females. These findings suggest that the TWBB population may not represent a significantly healthier cohort than the general population.

Conversely, the reduced cancer risk observed in the TWBB cohort might be attributed to the initial exclusion of all cancer patients. We selected comparisons that relied on NHI claim records that might not account for these potential cancer cases, resulting in elevated cancer risk among nonparticipants. However, this finding varied by cancer type. There were no significant differences in the risks for pancreatic, lung, kidney, hematologic, breast, or gynecological cancers between the groups. Notably, the risk for prostate cancer was higher in the TWBB cohort. The reasons for these variations remain unclear and may be related to underlying lifestyle behaviors or study sample size. This finding warrants further investigation.

It is important to note that TWBB may not fully represent the general population. Low participation rates in epidemiological studies can introduce bias, especially when there are systematic differences between participants and nonparticipants.^18^ However, if the study sample is large and exposures of interest vary sufficiently, results can still be generalized.^19^ For example, the United Kingdom Biobank, despite its low 5.5% response rate, showed similar mortality associations to studies with much higher response rates.^20^ To address discrepancies, weighted methods and post-stratification can align samples with target populations, as seen in studies adjusting for sociodemographic characteristics and risk factors. This approach can correct distortions, such as the initially observed protective association between alcohol use and cardiovascular disease mortality, which disappeared after post-stratification.^21^ Therefore, although TWBB may not be entirely representative, its emphasis on studying exposure-outcome associations renders its lack of representativeness less of a limitation. As with any observational study, researchers must remain vigilant in recognizing potential selection bias, modification effects, and residual confounding. These factors can influence the generalizability of exposure-outcome associations and should be carefully considered on a case-by-case basis.

One limitation of our study is the reliance on the NHI claims database for comparison purposes. Potential selection bias could affect our study results. For example, we could not exclude individuals who had already undergone evaluations for possible cancer. On the other hand, individuals with more medical conditions may be more concerned about health information and thus have a higher likelihood of being enrolled in the TWBB.^22^ Additionally, this database does not include lifestyle factors, such as alcohol consumption, smoking habits, or exercise levels, which could influence our findings. Whether these factors explain the differences in mortality rates and cancer incidence warrants further investigation. Furthermore, it is important to note that follow-up of the TWBB is ongoing. Monitoring further enrollees and re-examining the final results is imperative once the study is complete.

In summary, the TWBB cohort exhibits notable disparities in sociodemographic and health-related attributes compared to the general population. Predominantly, the TWBB cohort comprises older individuals and females with higher socioeconomic status, residing in urban areas. Notably, the all-cause mortality rate within the TWBB cohort exceeds that of the general Taiwanese population, while total cancer incidence rates are approximately 13% lower. Despite its lack of representativeness for deriving generalizable disease prevalence and incidence rates, the TWBB’s extensive size and diverse exposure measures offer valuable scientific insights into the associations between various exposures and health conditions.

## SUPPLEMENTARY MATERIAL

eTable 1. International Classification of Diseases (ICD) codes for health conditions

eTable 2. Distributions of sex, age, marital status, and education level among Taiwan Biobank participants in comparison with census data
