# Supplementary material for: Comparison of Demographic and Clinical Characteristics of Taiwan Biobank Participants With Nonparticipants
Source: J Epidemiol. 2025 Apr 5;35(4):206–11. doi: 10.2188/jea.JE20240297 (PMC11882352; doi:10.2188/jea.JE20240297)
Supplement: Supplementary file 1 [file je-35-206-s001.pdf]

eTable 1. International Classification of Diseases (ICD) codes for health conditions

	ICD-9	ICD-10
Cancer	140-208	C00-C97
Oropharyngeal cancer	140-149	C00-C10, C12-C14, C11
Esophageal cancer	150	C15
Stomach cancer	151	C16
Colorectal cancer	153, 154	C18, C19-C21
Liver cancer	155	C22
Pancreatic cancer	157	C25
Lung cancer	162	C33-C34
Breast cancer	174, 175	C50 (fifth code=1 or 2)
Gynecological cancer	179-184	C53-C54, C55, C58, C56-C574, C51-C52, C577-C579
Prostate cancer	185	C61
Bladder cancer	188	C67
Kidney cancer	189	C64-C66, C68
Hematologic malignancy	200-203, 205-208	C830, C833-C839, C846-C847, C852, C865-C866, C964, C965, C81, C802, C82, C831, C840-C841, C844, C849, C851, C858-C864, C884, C9140-C9142, C96, C88, C9001-C9032, C910-C9132, C915
Type I diabetes mellitus	250.x1, 250.x3	E10
Type II diabetes mellitus	250 (exclude 250.x1, 250.x3)	E08, E09, E11, E13
Hyperlipidemia	272	E78
Dementia	290, 294.1, 331.0-331.2	F00-F03, G30, G31.1
Substance use disorders	291, 292, 303.0, 303.9, 304, 305	F10-F19
Schizophrenia	295	F20, F25
Bipolar disorder	296.0-296.1, 296.4-296.8	F30, F31, F34.0,
Depressive disorder	296.2, 296.3, 300.4, 311	F32, F33, F34.1
Obsessive-compulsive disorder	300.3	F42
Anxiety disorder	300.0	F40, F41

Attention deficit-hyperactivity disorder	314	F90
Eating disorder	307.5, 307.1	F50
Somatoform	300.7, 300.8	F45
Adjustment reaction	309	F43
Parkinson's disease	332	G20
Multiple Sclerosis	340	G35
Epilepsy	345	G40
Migraine	346	G43
Extrapyramidal and movement disorders	332,333	G20-G26
Sleep apnea	327.2x	G47.3
Sleep disturbance	780.5x, 307.4x	G47.x, F51.x
Valvular heart disease	394-397, 424	I05-I08, I34-I37
Hypertension	401-405	I10, I11, I12, I13, I15
Coronary artery disease	410-414	I20-I25
Cardiomyopathy	425	I42-I43
Arrhythmia	427	I46.2, I46.8, I46.9, I47, I47.1, I47.2, I47.9, I48, I48.1, I48.2, I48.3, I48.4, I48.91, I48.92, I49.01, I49.02, I49.1, I49.2, I49.3, I49.4, I49.5, I49.8, I49.9
Stroke	430-438	I60-I69
Atrial fibrillation	427.31	I48.0, I48.2, I48.91
Chronic obstructive pulmonary disease	491, 492, 496	J41-J44
Asthma	493	J45
Gastroesophageal reflux disease	530.11, 530.81	K21
Peptic ulcer disease	531-534	K25-K28
Irritable bowel syndrome	564.1	K58
ulcerative colitis	556	K51
Crohn's disease	555	K50
Rheumatoid arthritis	714	M05-M06
Gout	274	M10

Osteoarthritis	715	M15-M19
Osteoporosis	733.0, 733.1	M80.00XA, M80.80XA, M84.40XA, M84.50XA, M84.60XA, M80.021A, M80.022A, M80.029A, M80.821A, M80.822A, M80.829A, M84.421A, M84.422A, M84.429A, M84.521A, M84.522A, M84.529A, M84.621A, M84.622A, M84.629A, M80.031A, M80.032A, M80.039A, M80.831A, M80.832A, M80.839A, M84.431A, M84.432A, M84.433A, M84.434A, M84.439A, M84.531A, M84.532A, M84.533A, M84.534A, M84.539A, M84.631A, M84.632A, M84.633A, M84.634A, M84.639A, M48.50XA, M48.51XA, M48.52XA, M48.53XA, M48.54XA, M48.55XA, M48.56XA, M48.57XA, M48.58XA, M80.08XA, M80.88XA, M84.48XA, M84.58XA, M84.68XA, M80.051A, M80.052A, M80.059A, M80.851A, M80.852A, M80.859A, M84.451A, M84.452A, M84.459A, M84.551A, M84.552A, M84.553A, M84.559A, M84.651A, M84.652A, M84.653A, M84.659A, M80.051A, M80.052A, M80.059A, M80.851A, M80.852A, M80.859A, M84.451A, M84.452A, M84.453A, M84.551A, M84.552A, M84.553A, M84.559A, M84.651A, M84.652A, M84.653A, M84.659A, M80.061A, M80.062A, M80.069A, M80.071A, M80.072A, M80.079A, M80.861A, M80.862A, M80.869A, M80.871A, M80.872A, M80.879A, M84.361A, M84.362A, M84.363A, M84.364A, M84.369A, M84.461A, M84.462A, M84.463A, M84.464A, M84.469A, M84.471A, M84.472A, M84.473A, M84.561A, M84.562A, M84.563A, M84.564A, M84.569A, M84.571A, M84.572A, M84.573A, M84.661A, M84.662A, M84.663A, M84.664A, M84.669A, M84.671A, M84.672A, M84.673A, M80.011A, M80.012A, M80.019A, M80.041A, M80.042A, M80.049A, M80.071A, M80.072A, M80.079A, M80.811A, M80.812A, M80.819A, M80.841A, M80.842A,

		M80.849A, M80.871A, M80.872A, M80.879A, M84.311A, M84.312A, M84.319A, M84.321A, M84.322A, M84.331A, M84.332A, M84.333A, M84.334A, M84.341A, M84.342A, M84.343A, M84.344A, M84.345A, M84.346A, M84.350A, M84.351A, M84.352A, M84.371A, M84.372A, M84.374A, M84.375A, M84.376A, M84.377A, M84.378A, M84.379A, M84.38xA, M84.411A, M84.412A, M84.419A, M84.441A, M84.442A, M84.443A, M84.444A, M84.445A, M84.446A, M84.454A, M84.474A, M84.475A, M84.476A, M84.477A, M84.478A, M84.479A, M84.48XA, M84.511A, M84.512A, M84.519A, M84.541A, M84.542A, M84.549A, M84.550A, M84.574A, M84.575A, M84.576A, M84.611A, M84.612A, M84.619A, M84.641A, M84.642A, M84.649A, M84.650A, M84.674A, M84.675A, M84.676A, M84.68XA, M81
Chronic kidney disease	585	N18
Renal stone	592, 594	N20-N22
Congenital heart disease	746	Q22-Q24
Self-harm behaviors	E950-E959	T1491, T360X2A, T361X2A, T362X2A, T363X2A, T364X2A, T365X2A, T365X4A, T366X2A, T367X2A, T368X2A, T3692XA, T370X2A, T371X2A, T372X2A, T373X2A, T374X2A, T375X2A, T378X2A, T3792XA, T380X2A, T381X2A, T382X2A, T383X2A, T384X2A, T385X2A, T386X2A, T387X2A, T38802A, T38812A, T38892A, T38902A, T38992A, T39012A, T39092A, T391X2A, T392X2A, T39312A, T39392A, T394X2A, T398X2A, T3992XA, T400X2A, T401X2A, T402X2A, T403X2A, T404X2A, T405X2A, T40602A, T40692A, T407X2A, T408X2A, T40902A, T40992A, T410X2A, T411X2A, T41202A, T41292A, T413X2A, T4142XA, T415X2A, T420X2A, T421X2A, T422X2A, T423X2A, T424X2A,

	T425X2A, T426X2A, T426X4A,<br>T4272XA, T428X2A, T43012A,<br>T43022A, T431X2A, T43202A, T43212A,<br>T43222A, T43292A, T433X2A,<br>T434X2A, T43502A, T43592A, T43602A,<br>T43612A, T43622A, T43632A, T43692A,<br>T438X2A, T4392XA, T440X2A,<br>T441X2A, T442X2A, T443X2A,<br>T444X2A, T445X2A, T446X2A,<br>T447X2A, T448X2A, T44902A,<br>T44992A, T450X2A, T451X2A,<br>T452X2A, T453X2A, T454X2A,<br>T45512A, T45522A, T45602A, T45612A,<br>T45622A, T45692A, T457X2A,<br>T458X2A, T4592XA, T460X2A,<br>T461X2A, T462X2A, T463X2A,<br>T464X2A, T465X2A, T466X2A,<br>T467X2A, T468X2A, T46902A,<br>T46992A, T470X2A, T471X2A,<br>T472X2A, T473X2A, T474X2A,<br>T475X2A, T476X2A, T477X2A,<br>T478X2A, T4792XA, T480X2A,<br>T481X2A, T48202A, T48292A,<br>T483X2A, T484X2A, T485X2A,<br>T486X2A, T48902A, T48992A,<br>T490X2A, T491X2A, T492X2A,<br>T493X2A, T494X2A, T495X2A,<br>T496X2A, T497X2A, T498X2A,<br>T4992XA, T500X2A, T501X2A,<br>T502X2A, T503X2A, T504X2A,<br>T505X2A, T506X2A, T507X2A,<br>T508X2A, T50902A, T50992A,<br>T50A12A, T50A22A, T50A92A,<br>T50B12A, T50B92A, T50Z12A,<br>T50Z92A, T510X2A, T511X2A,<br>T512X2A, T513X2A, T518X2A,<br>T5192XA, T520X2A, T521X2A,<br>T522X2A, T523X2A, T524X2A,<br>T528X2A, T5292XA, T530X2A,<br>T531X2A, T532X2A, T533X2A,<br>T534X2A, T535X2A, T536X2A,<br>T537X2A, T5392XA, T540X2A,<br>T541X2A, T542X2A, T543X2A,<br>T5492XA, T550X2A, T551X2A,<br>T560X2A, T561X2A, T562X2A,<br>T563X2A, T564X2A, T565X2A,<br>T566X2A, T567X2A, T56812A,<br>T56892A, T5692XA, T570X2A,<br>T571X2A, T572X2A, T573X2A,<br>T578X2A, T5792XA, T5802XA,

		T5812XA, T582X2A, T588X2A, T5892XA, T590X2A, T591X2A, T592X2A, T593X2A, T594X2A, T595X2A, T596X2A, T597X2A, T59812A, T59892A, T5992XA, T600X2A, T601X2A, T602X2A, T603X2A, T604X2A, T608X2A, T6092XA, T6102XA, T6112XA, T61772A, T61782A, T618X2A, T6192XA, T620X2A, T621X2A, T622X2A, T628X2A, T6292XA, T63002A, T63012A, T63022A, T63032A, T63042A, T63062A, T63072A, T63082A, T63092A, T63112A, T63122A, T63192A, T632X2A, T63302A, T63312A, T63322A, T63332A, T63392A, T63412A, T63422A, T63432A, T63442A, T63452A, T63462A, T63482A, T63512A, T63592A, T63612A, T63622A, T63632A, T63692A, T63712A, T63792A, T63812A, T63822A, T63832A, T63892A, T6392XA, T6402XA, T6482XA, T650X2A, T651X2A, T65212A, T65222A, T65292A, T653X2A, T654X2A, T655X2A, T656X2A, T65812A, T65822A, T65832A, T65892A, T6592XA, T71112A, T71122A, T71132A, T71152A, T71162A, T71192A, T71222A, T71232A, X710XXA, X711XXA, X712XXA, X713XXA, X718XXA, X719XXA, X72XXXA, X730XXA, X731XXA, X732XXA, X738XXA, X739XXA, X7401XA, X7402XA, X7409XA, X748XXA, X749XXA, X75XXXA, X76XXXA, X770XXA, X771XXA, X772XXA, X773XXA, X778XXA, X779XXA, X780XXA, X781XXA, X782XXA, X788XXA, X789XXA, X79XXXA, X80XXXA, X810XXA, X811XXA, X818XXA, X820XXA, X821XXA, X822XXA, X828XXA, X830XXA, X831XXA, X832XXA, X838XXA
Dysmenorrhea	625.3	N94.4, N94.5, N94.6
Uterine adenomyosis	617.0	N80.0
Endometriosis external	617.1-617.9	N80.1-N80.9
Uterine leiomyoma	218	D25

Polycystic ovary syndrome	256.4	E28.2	

ICD, International Classification of Diseases.

eTable 2. Distributions of sex, age, marital status, and education level among Taiwan Biobank participants in comparison with census data

	TWBB	2012 Census	2022 Census
Sex male, %	35.8	49.4	49.2
Age group, years, %			
30–39	23.0	29.8	24.8
40–49	24.2	28.3	27.0
50–59	29.6	26.5	25.9
60–70	23.2	15.5	22.3
Marital status, %			
Unmarried	14.0	18.2	23.0
Married	73.1	67.1	61.1
Divorced/separated	8.5	10.6	12.3
Death of spouse	4.4	4.1	3.6
Education, %			
Elementary school and below	5.1	14.7	5.1
Junior high school	7.3	18.1	15.5
Senior/vocational high school	29.0	45.8	43.6
College and above	58.6	21.3	35.7

TWBB, Taiwan Biobank.
